# Intra-individual variability of eGFR trajectories in early diabetic kidney disease and lack of performance of prognostic biomarkers

**DOI:** 10.1038/s41598-020-76773-0

**Published:** 2020-11-12

**Authors:** Julia Kerschbaum, Michael Rudnicki, Alexander Dzien, Christine Dzien-Bischinger, Hannes Winner, Hiddo Lambers Heerspink, László Rosivall, Andrzej Wiecek, Patrick B. Mark, Susanne Eder, Sara Denicolò, Gert Mayer

**Affiliations:** 1 Department of Internal Medicine IV (Nephrology and Hypertension), Medical University Innsbruck, Anichstrasse 35, 6020 Innsbruck, Austria; 2 Medical Center Hentschelhof, Innsbruck, Austria; 3 Department of Economics and Social Sciences, Paris-Lodron-University Salzburg, Salzburg, Austria; 4 Department of Clinical Pharmacy and Pharmacology, University of Groningen, University Medical Center Groningen, Groningen, The Netherlands; 5 International Nephrology Research and Training Centre, Institute of Translational Medicine, Semmelweis University, Budapest, Hungary; 6 Department of Nephrology, Transplantation and Internal Medicine, Medical University of Silesia, Katowice, Poland; 7 Institute of Cardiovascular and Medical Sciences, University of Glasgow, Glasgow, UK

**Keywords:** Medical research, Nephrology

## Abstract

Studies reporting on biomarkers aiming to predict adverse renal outcomes in patients with type 2 diabetes and kidney disease (DKD) conventionally define a surrogate endpoint either as a percentage of decrease of eGFR (e.g. ≥ 30%) or an absolute decline (e.g. ≥ 5 ml/min/year). The application of those study results in clinical practise however relies on the assumption of a linear and intra-individually stable progression of DKD. We studied 860 patients of the PROVALID study and 178 of an independent population with a relatively preserved eGFR at baseline and at least 5 years of follow up. Individuals with a detrimental prognosis were identified using various thresholds of a percentage or absolute decline of eGFR after each year of follow up. Next, we determined how many of the patients met the same criteria at other points in time. Interindividual eGFR decline was highly variable but in addition intra-individual eGFR trajectories also were frequently non-linear. For example, of all subjects reaching an endpoint defined as a decrease of eGFR by ≥ 30% between baseline and 3 years of follow up, only 60.3 and 45.2% lost at least the same amount between baseline and year 4 or 5. The results were similar when only patients on stable medication or subpopulations based on baseline eGFR or albuminuria status were analyzed or an eGFR decline of ≥ 5 ml/min/1.73m^2^/year was used. Identification of reliable biomarkers predicting adverse prognosis is a strong clinical need given the large interindividual variability of DKD progression. However, it is conceptually challenging in early DKD because of non-linear intra-individual eGFR trajectories. As a result, the performance of a prognostic biomarker may be accurate after a specific time of follow-up in a single population only.

## Introduction

Type 2 diabetes mellitus associated renal disease (diabetic kidney disease, DKD) is a serious public health problem and the leading cause of end stage renal disease (ESRD) in developed countries^1,2^. Nonetheless, when considering the large and continuously increasing number of patients at risk, only a fraction ultimately requires renal replacement therapy^3^. One explanation is the excessive competing risk of (mostly cardiovascular) mortality^4^, which increases in parallel to the decline in estimated glomerular filtration rate (eGFR)^5^. In addition, not all patients develop DKD^6–8^ and even those who do, progress at a highly variable rate^9^. The KDIGO guidelines suggest using eGFR and urinary albumin excretion (UAE) for cross sectional categorization of chronic kidney disease into 5 eGFR and 3 UAE stages^10^. On a cohort level this also provides reasonable information about the risk of DKD progression, defined by KDIGO either as a drop in eGFR category and a decline by at least 25% in eGFR or a loss of ≥ 5 ml/min/year/1.73m^2^^10^. However, personalized medicine mandates a more accurate risk prediction on the level of an individual^11–13^. In clinical practice this could trigger targeted therapy with an increased chance of success and/or a reduction of side effects^14^. Furthermore, recruitment of high-risk subjects into interventional studies at least theoretically allows decreasing the number of patients (and costs) necessary to reach statistically solid conclusions^15^.

Biomarkers are potentially excellent tools for describing subpopulations/individuals with different progression characteristics. Given the high clinical need and massive research activities invested, it is surprising that accurate prediction of prognosis in clinical practice is still difficult especially in early DKD and very few markers have undergone successful confirmatory testing in independent cohorts.

During the last decades it became evident that the pathophysiology of DKD is complex^16^. Next to systemic co-morbidities or effects of medication, the cross-sectional inter-^17^, and longitudinal intra-individual^18^ variability of pathways driving the disease leads to an unstable course over time^9^. This not only challenges the concept of a linear trajectory of eGFR decline but also conceptually creates a dilemma for biomarker research. To identify prognostic classifiers, observational or interventional studies are used, which follow patients for a specific period of time. Individuals are allocated to prognostic groups based on their progression characteristics (e.g. the decrease of eGFR) at the end of the observation period and baseline data are assessed to separate the strata. The internal and external validity of the findings however critically depend on the assumption that individuals remain in their category of prognosis irrespective of the further follow-up time. In case the course is not linear (e.g. based on variable pathophysiology and/or effects of therapy) we can expect patients changing their prognostic group, which will directly affect the accuracy of a prognostic biomarker (panel).

The objective of this study was to provide a descriptive analysis of the intra-individual variability in eGFR trajectories in early DKD. Common surrogate endpoints used in published biomarker studies (for example in^19–22^ on DKD progression were examined regarding their stability over 5 years of follow-up.

We analyzed data from two independent prospective cohorts. Based on various definitions of the change in eGFR between baseline and a specific point during clinical follow- up, patients were allocated to a group with a detrimental prognosis. Next, we evaluated how many of these individuals met the respective definition also at other time points of follow-up. In case of a stable eGFR trajectory the group with a detrimental prognosis should be composed of the same individuals over time whereas intra-individual non-linearity of progression will result in the contrary.

## Results

Details for participants are presented in supplementary Table S1. Of note, mean baseline eGFR was preserved in both groups and decreased by approximately 7 ml/min/1.73m^2^ during the follow up period.

To analyze the intra-individual variability of eGFR decline, we used the two-point method focusing on the percentage of the change of eGFR between baseline and each follow-up visit. Of the 860 individuals selected from the PROVALID study, all patients who had a decline of eGFR between baseline and each individual year of follow up of ≥ 25, ≥ 30, ≥ 35 or ≥ 40% were identified. Results for the threshold of ≥ 30% eGFR decline are given in Table 1, all other (similar) results in supplementary Table S2. The number of individuals reaching the endpoint increased continuously from 25 at a one year to 117 at a year 5 comparison, which is compatible with the concept that DKD is a progressive disease on a cohort level. On an individual basis, eGFR trajectories were non-linear in many subjects. For example, 73 patients lost more than 30% of eGFR between baseline and follow-up 3. Of these, only 8 (10.9%) and 29 (39.7%) already met the definition after 1 and 2 years, which could be explained by slower progression in the rest. However, after 4 and 5 years, only 44 (60.3%) and 33 (45.2%) of these individuals again were grouped in this category, the remainder obviously partially recovered eGFR after year 3. In the validation cohort identical results were obtained (bottom Table 1), but the number of subjects was smaller.

**Table 1:** Intra-individual stability of an eGFR decline ≥ 30% over time.

	eGFR decline ≥ 30% from baseline until				
	1st year of FU	2nd year of FU	3rd year of FU	4th year of FU	5th year of FU
**PROVALID (n = 860)**					
FU1 (n/%)	25 (100)	**6 (24.0)**	**4 (16.0)**	**4 (16.0)**	**4 (16.0)**
FU2 (n/%)	6 (10.9)	55 (100)	**29 (52.7)**	**20 (36.4)**	**16 (29.1)**
FU3 (n/%)	8 (11.0)	29 (39.7)	73 (100)	**44 (60.3)**	**33 (45.2)**
FU4 (n/%)	15 (16.0)	26 (27.7)	44 (46.8)	94 (100)	**53 (56.4)**
FU5 (n/%)	14 (12.0)	32 (27.4)	44 (37.6)	53 (45.3)	117 (100)
**Validation cohort (n = 178)**					
FU1 (n/%)	5 (100)	**4 (80.0)**	**2 (40.0)**	**2 (40.0)**	**2 (40.0)**
FU2 (n/%)	4 (36.4)	11 (100)	**5 (45.5)**	**4 (36.4)**	**4 (36.4)**
FU3 (n/%)	2 (33.3)	5 (83.3)	6 (100)	**4 (66.7)**	**4 (66.7)**
FU4 (n/%)	4 (20.0)	5 (25.0)	4 (20.0)	20 (100)	**13 (65.0)**
FU5 (n/%)	4 (16.0)	7 (28.0)	6 (24.0)	13 (52.0)	25 (100)

*FU* follow up.

The tables should be read as follows: The vertical lines indicate the number and percentage of patients meeting a specific definition of eGFR decline over time. For example, 25 individuals of the PROVALID cohort had a decrease of eGFR ≥ 30% after one year of follow-up and form the cohort that is followed (100%). Of these, only 6 meet also meet the definition of eGFR decline after 2 years of follow-up (24%) (these numbers are given in bold letters). In the next line, we used a definition of a decline in eGFR ≥ 30% during the first 2 years of follow-up and identified 55 individuals (again forming 100% of the population). Of these, only 6 (10.9%) already have lost more than 30% of baseline eGFR after one year, whereas 26 individuals recovered renal function during the third year of follow-up, leaving only 29 (52.7%) individuals persistently meeting the definition of eGFR decline. When looking at the diagonal reading, one can see that on a cohort level the number of patients with a loss of eGFR ≥ 30% is increasing from 25 to 117 over time.

To evaluate if non-linearity of the eGFR trajectories was induced by changes in drug prescriptions, we defined a subset of PROVALID participants in whom neither treatment with RAAS blocking agents nor SGLT-2 inhibitors was introduced or discontinued during the entire period (n = 552), but similar results were obtained (data for a threshold of ≥ 30% eGFR decline are given on top of supplementary Table S3, details on all other thresholds in supplementary Table S4). Even when the analysis was restricted to those individuals, who also had a stable prescription of calcium antagonists, diuretics, and non-steroidal anti-inflammatory drugs (n = 277), results did not change substantially (bottom supplementary Table S3, but the number of individuals analysed became low.

Many clinical trials use the definition of a “confirmed reduction in eGFR” and we thus looked into the PROVALID cohort to evaluate the impact of allocating only those patients to the group with a detrimental prognosis, who had a reduction of ≥ 30% between baseline at two consecutive time points during follow-up. As shown for example in Table 2, 29 patients had a reduction in eGFR of ≥ 30% at the second follow-up which was confirmed in the third follow-up. Of these however, again only 20 (69%) and 16 (55.2%) also met this endpoint after 4 and 5 years.

**Table 2:** Intra-individual stability of a “confirmed” eGFR decline ≥ 30% over time.

n = 860	Confirmed eGFR decline ≥ 30% from baseline until			
	1st and 2nd FU	3rd FU	4th FU	5th FU
n (%)	6 (100%)	**4 (66.7%)**	**4 (66.7%)**	**4 (66.7%)**
	1st FU	2nd and 3rd FU	4th FU	5th FU
n (%)	4 (13.8%)	29 (100%)	**20 (69.0%)**	**16 (55.2%)**
	1st FU	2nd FU	3rd and 4th FU	5th FU
n (%)	4 (9.1%)	20 (45.5%)	44 (100%)	**33 (75.0%)**
	1st FU	2nd FU	3rd FU	4th and 5th FU
n (%)	4 (7.5%)	16 (30.2%)	33 (62.3%)	53 (100%)

*FU* follow up.

Bold numbers: number of individuals (%) persistently meeting the definition of eGFR decline over time.

Finally, we defined “rapid progression” as a loss of eGFR of ≥ 5 ml/min/1.73m^2^/year of follow up (slope method). Table 3 shows that this approach also did not define a consistent group over time. For example, 206 individuals of the PROVALID cohort experienced this eGFR decline at follow-up 3, but only 119 (57.8%), and 80 (38.8%) also met the definition at follow-up 4 and 5. Results were again similar in the validation cohort (bottom Table 3) and for the 552 individuals on stable RAAS and SGLT-2 inhibitor therapy (supplementary Table S5).

**Table 3:** Intra-individual stability of an eGFR decline ≥ 5 ml/min/1.73m^2^/year over time.

	eGFR decline ≥ 5 ml/min/1.73m^2^/year until			
	2nd year of FU	3rd year of FU	4th year of FU	5th year of FU
**PROVALID (n = 860)**				
FU2 (n/%)	288 (100)	**169 (58.7)**	**112 (38.9)**	**79 (27.4)**
FU3 (n/%)	169 (82.0)	206 (100)	**119 (57.8)**	**80 (38.8)**
FU4 (n/%)	112 (76.2)	119 (81.0)	147 (100)	**87 (59.2)**
FU5 (n/%)	79 (74.5)	80 (75.5)	87 (82.1)	106 (100)
**Validation cohort (n = 178)**				
FU2 (n/%)	46 (100)	**19 (41.3)**	**12 (26.1)**	**10 (21.7)**
FU3 (n/%)	19 (79.2)	24 (100)	**13 (54.2)**	**10 (41.7)**
FU4 (n/%)	12 (63.2)	13 (68.4)	19 (100)	**15 (78.9)**
FU5 (n/%)	10 (55.6)	10 (55.6)	15 (83.3)	18 (100)

*FU* follow up.

Bold numbers: number of individuals (%) persistently meeting the definition of eGFR decline over time. The calculation of the slopes is based on linear regressions using at least 3 eGFR observations for each patient. For this reason, a comparison between baseline and FU1 is missing in the table.

To evaluate the impact of baseline eGFR and albuminuria on the individual stability of an eGFR decline ≥ 30% or ≥ 5 ml/min/1.73m^2^/year, we divided participants of the PROVALID cohort into categories of baseline eGFR (≥ 60 and < 60 ml/min/1.73m^2^) or baseline urinary albumin-to-creatinine ratios (UACR, < 30 or ≥ 30 mg/g). Results did not change significantly (supplementary Tables S6, S7, S8 and S9), however with small numbers of individuals in the groups.

## Discussion

The rate of progression varies between individuals in chronic kidney disease^10^ and especially in DKD^23^. This observation stimulates research to develop biomarkers able to replace the current albuminuria and eGFR based cohort centric probabilistic approach of estimating prognosis by a more deterministic assessment at the level of an individual.

One crucial element in implementation of biomarkers is the definition of the endpoint to be predicted. Choosing the incidence of ESRD and/or a doubling serum creatinine is reasonable in more advanced stages of DKD, but not absolutely preferred in early phases given the prolonged time of follow up necessary, leaving ample room for competing risks. As an alternative, the KDIGO guidelines suggest a decrease of eGFR exceeding 25% or a loss of more than 5 ml/min/1.73m^2^/year to define patients at highest risk and their early identification becomes a more realistic aim. Biomarker research in this area conventionally relies on information from observational cohorts or interventional trials and allocates individuals to a high risk/fast progression group. Next, baseline data are analysed to define a biomarker/a biomarker panel that separates these individuals from controls/individuals with slow progression. Unfortunately, the success to predict changes in eGFR by biomarkers hitherto is limited. One explanation is their often limited, additional information when used on top of albuminuria and eGFR. This could be a consequence of a bad markers selection and much effort focuses on “Omics”-profiling and machine learning techniques to advance, but again only little progress has been achieved so far and the cost effectiveness remains to be determined^24^. In addition, based on our results, we postulate that the failure to identify accurate and reliable prognostic biomarkers to decipher inter-individual heterogeneity in DKD progression is due to a lack of studies to acknowledge intra-individual variability in progression. Supplementary Tables S10a,b show a summary of biomarker studies in type 2 diabetes assessed via Pubmed in June, 2019. For example, Saulnier et al.^19^ explored the prognostic value of midregional-proadrenomedullin (MR-proADM), soluble tumor necrosis factor receptor 1 (sTNFR1), and N-terminal prohormone brain natriuretic peptide (NT-proBNP) for a decline in eGFR of ≥ 40% during 4.3 years of follow-up or an eGFR annual slope ≥ 5 ml/min/1.73m^2^/year. Mise et al.^20^ analysed the association of urinary levels of glycans binding to six lectins in 675 participants over a follow-up period of 4.0 years and a decrease in eGFR of ≥ 30% or dialysis. Chung et al.^21^ evaluated the predictive value of n-3 polyunsaturated fatty acids and interleukin-6 in 676 participants with type 2 diabetes. Renal function decline was defined as an eGFR decline of ≥ 25% over a 4-year period. In a sub-analysis of the Nurses’ Health Study^22^, the association of soluble tumour necrosis factor receptor 2 and an eGFR decline of ≥ 25% over 11 years was evaluated. Most of those studies could show a significant association of the analysed biomarker and the renal endpoint, but if the patients meeting the defined endpoint are not the same at different points of follow up any biomarker defining the group at a specific point in time loses precision at others.

As described above, in our study we were unable to define a group of individuals that over time stably met the definition of a poor outcome despite using various, guideline approved endpoint definitions (even a “confirmed drop of eGFR”) (Table 4). Variability in intra-individual progression has also been reported by others. For example, in the African American Study of Kidney Disease 41.6% of participants exhibited a greater than 90% probability of having a non-linear trajectory; in 66.1% the probability of non-linearity was > 50%^25^. We do not suggest that an eGFR slope or a percentage drop of eGFR are not valid surrogate endpoints for kidney disease progression in clinical trials on a cohort level as recently shown by Inker et al.^26^, but rather argue that their prognostic information content for an individual in early DKD is low. This finding does not preclude better performance of markers in later stages of DKD, as the trajectories of renal function loss may be much more linear towards end-stage renal disease.

**Table 4:** Comparison of different methods to assess the individual stability over time of an eGFR decline from baseline until FU 3.

PROVALID (n = 860)	≥ 30% decline from baseline	≥ 5 ml/min/1.73m^2^/year	Confirmed reduction ≥ 30% in 2 consecutive FUs
FU 2, n (%)	29 (39.7)	169 (82.0)	4 (13.8)
FU 3, n (%)	73 (100)	206 (100)	29 (100)
FU 4, n (%)	**44 (60.3)**	**119 (57.8)**	**20 (69.0)**
FU 5, n (%)	**33 (45.2)**	**80 (38.8)**	**16 (55.2)**

*FU* follow up.

Bold numbers: number of individuals (%) persistently meeting the definition of eGFR decline over time.

For example, a set of nine biomarkers was selected based on pathophysiological reasoning and measured in baseline samples of 1,765 patients recruited into two clinical trials^27^. The variability of the annual loss of eGFR explained by the biomarkers, indicated by the adjusted R^2^ value, was 15% and 34% for patients with eGFR ≥ 60 and < 60 ml/min/1.73 m^2^, respectively; variability explained by clinical predictors was 20% and 31% and a combination of both increased the adjusted R^2^ to 35% and 64%. In summary, predicting the individual eGFR slope seems more feasible in advanced disease but is difficult in individuals with the greatest clinical need, i.e. those with a (relatively) preserved renal function.

Interestingly, our results did not change substantially when we analysed only individuals on stable medication. This is a strong indication that the efficacy of interventions also is variable over time. If confirmed in other cohorts this observation not only has to be taken into account when developing biomarkers predicting treatment response. It will also mandate a critical appraisal of the current clinical practise of caring for patients with DKD as re-assessment of prognosis as well as efficacy of therapy will be needed in regular intervals. This conclusion is supported by a publication of Zewinger et al. Measurements of urinary Dickkopf-3 levels, a stress-induced tubular epithelia-derived pro-fibrotic glycoprotein, in patients with IgA nephropathy predicted eGFR decline for the next a maximum of 6 months but, if repeated, also over a prolonged period of time^28^.

It could be argued that our results are caused by fluctuations of serum creatinine levels that are not due to changes in renal function. However, Hilderink et al. showed that this spontaneous variability leads to fluctuations of eGFR of 13–20%^29^ only, a value lower than many of our criteria used to define a group of individuals with a detrimental prognosis. Nonetheless we could not evaluate the stability of an eGFR decline of ≥ 45 or 50% as the patient number became low. This however is a clear indication of a limited clinical practise relevance of this definition in early DKD with relatively preserved eGFR. A clear limitation of our study is that the number of individuals in our eGFR or albuminuria subgroup analysis is small, but in any case, the results are consistent with the other analyses.

Available biomarker studies posthoc defined groups of progressors and non-progressor (inter-individual variability) based on either a defined slope of eGFR over time or a percentage of decrease of eGFR over a period of 2–5 years. Next, biomarkers were analyzed at baseline to discriminate between the two groups. With this approach, adjustment for other covariates at baseline to evaluate the performance of the biomarkers is definitely helpful. However, we focus on intra-individual longitudinal variability in progression. This phenomenon questions the validity of the endpoint and adjustment for baseline factors is not able to handle the problem. However, we did a subgroup analysis with patients stratified for albuminuria status at baseline, the most prominent risk factor for progression in DKD. As can be seen in supplementary Table S8 the intra-individual variability in the changes in eGFR was independent of albuminuria status at baseline. Therefore, we can conclude that the stability of the intra-individual change in eGFR over time does not depend on the magnitude of baseline albuminuria.

In summary, our study shows that in early DKD progression, when defined by a (confirmed or non-confirmed) decline in eGFR of ≥ 25, ≥ 30, ≥ 35 or ≥ 40% or a loss of ≥ 5 ml/min/1.73m^2^/year at any point in time within a period of 5 years, does not reliably identify individual patients that form a stable group with high risk. Therefore, biomarkers predicting the individual risk at a specific point in time in a study are not necessarily also accurate at other points in time or in independent cohorts.

## Methods

### Study population

For evaluating the decline of eGFR in type 2 diabetes we used data from patients recruited into the “Prospective cohort study in patients with type 2 diabetes mellitus for validation of biomarkers” (PROVALID). Details of the trial are presented elsewhere^30^. In summary, the PROVALID database provides annual information on medication and the incidence and progression of renal and cardiovascular disease in 4000 prevalent patients with type 2 diabetes in five European countries (Austria, Hungary, the Netherlands, Poland and Scotland) all being taken care at the primary level of healthcare. We selected 860 individuals with complete annual follow up information for at least 5 consecutive years. Exclusion criteria were an age < 18 years, a BMI < 18 or > 40 kg/m^2^ and a baseline eGFR value > 150 ml/min/1.73m^2^. Serum creatinine was determined by the IDMS traceable methodology and eGFR was calculated by the CKD-EPI equation^31^.

In addition, we had access to a cohort of 768 patients with type 2 diabetes, who attended a medical practice in the area of Innsbruck between January 1995 and April 2018 (validation cohort). Further details on this population are given in^32^. We used the same in- and exclusion criteria as described above and identified 178 individuals. As these patients were not included into a study with fixed follow up schedules, the mean time between the visits was 1.29 years and the mean total follow up time was 7.7 ± 2.0 years.

For the analysis of variability in eGFR decline we used gender and age for calculation of eGFR, and albuminuria status and medication (RAAS inhibitors, SGLT2 inhibitors, calcium antagonists, diuretics and non steroidal anti-inflammatory agents) for stratification.

The PROVALID study and sub-analyses of the study were approved by Institutional Review Boards in all participating countries (Austria: Ethics committee of the Medical University Innsbruck, Ethics committee of Upper Austria; Poland: Ethics Committee of the Medical University of Silesia; the Netherlands: Medical Ethical Committee of the University Medical Center Groningen (UMCG); United Kingdom: NHS Research Ethics Committee; Hungary: Semmelweis University, Department of Bioethics). In the validation cohort an informed consent about the participation was provided by all patients. All study-related interventions were conducted in accordance with the Declaration of Helsinki and Good Clinical Practice.

### Statistics

Surrogate endpoints used to analyze the intra-individual variability in eGFR decline descriptively were the same as they were used in a majority of biomarker studies on this topic (supplemental tables S10a,b): (1) a percentage decline of eGFR between baseline and each consecutive follow-up visit of more than 25, 30, 35 or 40% (two-point method) or (2) a loss of eGFR of ≥ 5 ml/min/1.73m^2^/year (slope method). Regarding (2), we applied a standard linear regression model for each individual. In particular, we regressed the individual eGFR on a time trend (indication each follow-up measurement) and stored the slope coefficient of each regression.

We next calculated the number of individuals reaching the eGFR surrogate endpoint at a specific year of follow up and the percentage of these individuals, that also met the same surrogate endpoint at other follow up time points.

As many more recent biomarker studies also use a “confirmed” change in eGFR (loss of a specific amount of eGFR at one and another consecutive measurement) we also applied this criterion in a sub-analysis.

To eliminate the impact of changes of treatment on eGFR trajectories we also performed all analyses in the PROVALID cohort in a subpopulation of 552 patients, who were receiving or not receiving renin-angiotensin aldosterone system (RAAS) blocking agents and SGLT-2 inhibitors during the entire follow up period and in 277 individuals in whom also other medications (e.g. diuretics, calcium channel blockers or NSAIDS) were unaltered. In addition, a separate analysis was performed in individuals based on baseline eGFR (< 60 and ≥ 60 ml/min/1.73m^2^) and albuminuria status (normo- or micro- and macroalbuminuria).

Finally, the comparison of characteristics between the PROVALID and the validation cohort reported in supplementary Table S1 is based on an unpaired two-sample t-test on equal means. The p-value in the last column is based on a two-sided test. All the empirical analysis is carried out with Stata (version 16).

## Supplementary information

Supplementary Information

## Data Availability

The data that support the findings of this study are available from the PROVALID investigators and from the Medical Center Hentschelhof, but restrictions apply to the availability of these data, which were used under license for the current study, and so are not publicly available. Data are however available from the authors upon reasonable request and with permission of the PROVALID investigators and the Medical Center Hentschelhof.
