# Supplementary material for: Intra-individual variability of eGFR trajectories in early diabetic kidney disease and lack of performance of prognostic biomarkers
Source: Sci Rep. 2020 Nov 12;10:19743. doi: 10.1038/s41598-020-76773-0 (PMC7665005; doi:10.1038/s41598-020-76773-0)
Supplement: Supplementary file 1 — Supplementary Information [file 41598_2020_76773_MOESM1_ESM.pdf]

Supplemental material to

INTRA-INDIVIDUAL VARIABILITY OF eGFR TRAJECTORIES IN EARLY DIABETIC KIDNEY DISEASE AND LACK OF PERFORMANCE OF PROGNOSTIC BIOMARKERS

Julia Kerschbaum[1*], MD, Michael Rudnicki[1], MD, Alexander Dzien[2], MD, Christine Dzien-Bischinger[2], MD, Hannes Winner[3], PhD, Hiddo Lambers Heerspink[4], PhD, László Rosivall[5], PhD, Andrzej Wiecek[6], MD, PhD, Patrick B. Mark, PhD, Susanne Eder[1], PhD, Sara Denicolò[1], MD, Gert Mayer[1], MD

[1] Department of Internal Medicine IV (Nephrology and Hypertension), Medical University Innsbruck, Innsbruck, Austria

[2] Medical Center Hentschelhof, Innsbruck, Austria

[3] Department of Economics and Social Sciences, Paris-Lodron-University Salzburg, Salzburg, Austria

[4] Department of Clinical Pharmacy and Pharmacology, University of Groningen, University Medical Center Groningen, Groningen, The Netherlands

[5] International Nephrology Research and Training Centre, Institute of Translational Medicine, Semmelweis University, Budapest, Hungary

[6] Department of Nephrology, Transplantation and Internal Medicine, Medical University of Silesia, Katowice, Poland

[7] Institute of Cardiovascular & Medical Sciences, University of Glasgow, Glasgow, UK

Corresponding author:

Julia Kerschbaum, MD, MSc, MPH

Department of Internal Medicine (Nephrology and Hypertension)

Medical University Innsbruck

Anichstrasse 35

6020 Innsbruck

Austria

Tel.: 0043 512 504 81333

Fax: 0043 512 504 25857

Email: julia.kerschbaum@i-med.ac.at

Supplemental materials

Supplementary table 1

Characteristics of the study populations

	PROVALID cohort (n = 860)	Validation cohort (n = 178)	p-value
Baseline age (years)	64.0 ± 9.6	66.3 ± 11.4	0.005
Males (n/%)	471 (54.8)	91 (51.1)	0.375
Baseline eGFR (ml/min/1.73m^2)	81.8 ± 23.1	72.5 ± 16.7	0.001
eGFR last follow up (ml/min/1.73m^2)	74.6 ± 22.9	65.7 ± 18.2	0.001
Mean duration of diabetes at baseline (years)	10.9 ± 8.3	6.5 ± 5.9	0.001
Systolic blood pressure at baseline (mmHg)	136.5 ± 16.4	145.5 ± 17.6	0.001
Diastolic blood pressure at baseline (mmHg)	79.1 ± 9.6	85.5 ± 8.4	0.001
BMI at baseline (kg/m^2)	30.1 ± 4.1	27.8 ± 4.1	0.001
HbA1c at baseline (%)	6.9 ± 1.0	6.4 ± 1.0	0.001
stable on RAS blocking therapy during follow up (%)	77.1	80.3	0.343
Prevalent heart failure at baseline (%)	2.2	6.2	0.004
Prevalent other CV or peripheral arterial disease at baseline (%) †	18.4	26.4	0.014
Prevalent cerebrovascular disease at baseline (%) ‡	6.6	6.2	0.826

† history of myocardial infarction or coronary revascularization.
‡ history of stroke or TIA

Supplementary table 2

Intra-individual stability of an eGFR decline ≥25, ≥35 or ≥40 over time

	eGFR decline ≥25% from baseline until				
	1st year of FU	2nd year of FU	3rd year of FU	4th year of FU	5th year of FU
PROVALID (n=860)					
FU1 (n/%)	51 (100)	**25 (49.0)**	**17 (33.3)**	**14 (27.5)**	**12 (23.5)**
FU2 (n/%)	25 (25.3)	99 (100)	**54 (54.5)**	**42 (42.4)**	**39 (39.4)**
FU3 (n/%)	27 (21.3)	54 (42.5)	127 (100)	**82 (64.6)**	**67 (52.8)**
FU4 (n/%)	27 (18.0)	55 (36.7)	82 (54.7)	150 (100)	**98 (65.3)**
FU5 (n/%)	33 (19.0)	63 (36.2)	80 (46.0)	98 (46.0)	174 (100)
	eGFR decline ≥35% from baseline until				
	1st year of FU	2nd year of FU	3rd year of FU	4th year of FU	5th year of FU
PROVALID (n=860)					
FU1 (n/%)	10 (100)	**2 (20.0)**	**2 (20.0)**	**2 (20.0)**	**2 (20.0)**
FU2 (n/%)	2 (5.7)	35 (100)	**17 (48.6)**	**11 (31.4)**	**10 (28.6)**
FU3 (n/%)	5 (8.3)	17 (35.4)	48 (100)	**29 (60.4)**	**21 (43.8)**
FU4 (n/%)	8 (13.8)	12 (20.7)	29 (50.0)	58 (100)	**30 (51.7)**
FU5 (n/%)	7 (9.9)	17 (23.9)	29 (40.8)	40 (42.3)	71 (100)
	eGFR decline ≥40% from baseline until				
	1st year of FU	2nd year of FU	3rd year of FU	4th year of FU	5th year of FU
PROVALID (n=860)					
FU1 (n/%)	5 (100)	**1 (20.0)**	**1 (20.0)**	**1 (20.0)**	**1 (20.0)**
FU2 (n/%)	1 (5.3)	19 (100)	**8 (42.1)**	**5 (26.3)**	**4 (21.1)**
FU3 (n/%)	3 (12.0)	8 (32.0)	25 (100)	**14 (56.0)**	**9 (36.0)**
FU4 (n/%)	4 (11.1)	9 (25.0)	14 (38.9)	36 (100)	**20 (55.6)**
FU5 (n/%)	5 (11.9)	10 (23.8)	15 (35.7)	20 (47.6)	42 (100)

FU: follow up; bold numbers: Number of individuals (%) persistently meeting the definition of eGFR decline over time

Supplementary table 3

Intra-individual stability of an eGFR decline ≥30% of patients on stable medication in the PROVALID study over time

	eGFR decline ≥30% from baseline until				
	1st year of FU	2nd year of FU	3rd year of FU	4th year of FU	5th year of FU
RAAS inhibitors and SGLT2 inhibitors stable (PROVALID) (n=552)					
FU1 (n/%)	18 (100)	**5 (27.8)**	**3 (16.7)**	**3 (16.7)**	**3 (16.7)**
FU2 (n/%)	5 (13.2)	38 (100)	**19 (50.0)**	**14 (36.8)**	**12 (31.6)**
FU3 (n/%)	5 (10.4)	19 (39.6)	48 (100)	**30 (62.5)**	**23 (47.9)**
FU4 (n/%)	10 (16.7)	18 (30.0)	30 (50.0)	60 (100)	**35 (58.3)**
FU5 (n/%)	11 (15.3)	22 (30.6)	29 (40.3)	35 (48.6)	72 (100)
	eGFR decline ≥30% from baseline until				
	1st year of FU	2nd year of FU	3rd year of FU	4th year of FU	5th year of FU
RAAS inhibitors, SGLT2 inhibitors, calcium antagonists, diuretics and non steroidal anti-inflammatory agents stable (n=277)					
FU1 (n/%)	6 (100)	**2 (33.3)**	**1 (16.7)**	**1 (16.7)**	**1 (16.7)**
FU2 (n/%)	2 (11.1)	18 (100)	**9 (50.0)**	**8 (44.4)**	**7 (38.9)**
FU3 (n/%)	1 (6.3)	9 (56.3)	16 (100)	**10 (62.5)**	**8 (50.0)**
FU4 (n/%)	1 (3.7)	10 (37.0)	10 (37.0)	27 (100)	**13 (48.1)**
FU5 (n/%)	4 (13.8)	11 (37.9)	9 (31.0)	13 (44.8)	29 (100)

FU: follow up; bold numbers: Number of individuals (%) persistently meeting the definition of eGFR decline over time

Supplementary table 4

Intra-individual stability of an eGFR decline ≥25%, ≥35% and ≥40% for the PROVALID cohort on stable RAAS blocking and SGLT-2 inhibitor therapy over time

	eGFR decline ≥25% from baseline until				
	1st year of FU	2nd year of FU	3rd year of FU	4th year of FU	5th year of FU
PROVALID (n=552)					
FU1 (n/%)	35 (100)	**21 (60.0)**	**13 (37.1)**	**10 (28.6)**	**8 (22.9)**
FU2 (n/%)	21 (33.3)	63 (100)	**34 (54.0)**	**26 (41.3)**	**23 (36.5)**
FU3 (n/%)	19 (23.5)	34 (42.0)	81 (100)	**55 (67.9)**	**45 (55.6)**
FU4 (n/%)	18 (19.8)	34 (36.4)	55 (60.4)	91 (100)	**61 (67.0)**
FU5 (n/%)	21 (19.1)	40 (36.4)	53 (48.2)	61 (55.5)	110 (100)
	eGFR decline ≥35% from baseline until				
	1st year of FU	2nd year of FU	3rd year of FU	4th year of FU	5th year of FU
PROVALID (n=552)					
FU1 (n/%)	6 (100)	**1 (16.7)**	**1 (16.7)**	**1 (16.7)**	**1 (16.7)**
FU2 (n/%)	1 (4.3)	23 (100)	**10 (43.5)**	**9 (39.1)**	**8 (34.8)**
FU3 (n/%)	2 (6.7)	10 (33.3)	30 (100)	**21 (70.0)**	**15 (50.0)**
FU4 (n/%)	4 (10.3)	10 (25.6)	21 (53.8)	39 (100)	**22 (56.4**)
FU5 (n/%)	4 (9.3)	12 (27.9)	18 (41.9)	22 (51.2)	43 (100)
	eGFR decline ≥40% from baseline until				
	1st year of FU	2nd year of FU	3rd year of FU	4th year of FU	5th year of FU
PROVALID (n=552)					
FU1 (n/%)	2 (100)	**1 (0.0)**	**1 (50.0)**	**1 (50.0)**	**1 (50.0)**
FU2 (n/%)	1 (8.3)	12 (100)	**4 (33.3)**	**4 (33.3)**	**3 (25.0)**
FU3 (n/%)	2 (11.8)	4 (23.5)	17 (100)	**11 (64.7)**	**8 (47.1)**
FU4 (n/%)	2 (8.0)	8 (32.0)	11 (44.0)	25 (100)	**16 (64.0**)
FU5 (n/%)	2 (7.1)	7 (25.0)	10 (35.7)	16 (57.2)	28 (100)

FU: follow up; bold numbers: Number of individuals (%) persistently meeting the definition of eGFR decline over time

Supplementary table 5

Intra-individual stability of an eGFR decline ≥5 ml/min/1.73m^2/year in a cohort of patients with stable RAAS and SGLT-2 inhibitor therapy in the PROVALID study over time

	eGFR decline ≥5ml/min/1.73m^2/year			
	2nd year of FU	3rd year of FU	4th year of FU	5th year of FU
PROVALID (n=552)				
FU2 (n/%)	184 (100)	**107 (58.2)**	**69 (37.5)**	**49 (26.6)**
FU3 (n/%)	107 (83.6)	128(100)	**73 (57.0)**	**52 (40.6)**
FU4 (n/%)	69 (82.1)	73 (86.9)	84(100)	52 (61.9)
FU5 (n/%)	49 (76.6)	52 (81.3)	52 (81.3)	64 (100)

FU: follow up; bold numbers: Number of individuals (%) persistently meeting the definition of eGFR decline over time. The calculation of the slopes is based on linear regressions using at least 3 eGFR observations for each patient. For this reason, a comparison between baseline and FU1 is missing.

Supplementary table 6

Intra-individual stability of an eGFR decline ≥30% in different eGFR-cohorts in the PROVALID population over time

	eGFR decline ≥30% from baseline until				
	1st year of FU	2nd year of FU	3rd year of FU	4th year of FU	5th year of FU
eGFR < 60 ml/min (n=149)					
FU1 (n/%)	4 (100)	**1 (25.0)**	**1 (25.0)**	**1 (25.0)**	**1 (25.0)**
FU2 (n/%)	1 (9.1)	11 (100)	**7 (63.5)**	**4 (36.4)**	**4 (36.4)**
FU3 (n/%)	2 (11.8)	7 (41.2)	17 (100)	**10 (58.8)**	**8 (47.1)**
FU4 (n/%)	3 (20.0)	4 (26.7)	10 (66.7)	15 (100)	**11 (73.3)**
FU5 (n/%)	3 (18.8)	7 (43.8)	10 (62.5)	11 (68.8)	16 (100)
eGFR ≥ 60 ml/min (n=711)					
FU1 (n/%)	21 (100)	**5 (23.8)**	**3 (14.3)**	**3 (14.3)**	**3 (14.3)**
FU2 (n/%)	5 (11.4)	44 (100)	**22 (50.0)**	**16 (36.4)**	**12 (27.3)**
FU3 (n/%)	6 (10.7)	22 (39.3)	56 (100)	**34 (60.7)**	**25 (44.6)**
FU4 (n/%)	12 (15.2)	22 (27.8)	34 (43.0)	79 (100)	**42 (53.2)**
FU5 (n/%)	11 (10.9)	25 (24.8)	34 (33.7)	42 (41.6)	101 (100)

FU: follow up; bold numbers: Number of individuals (%) persistently meeting the definition of eGFR decline over time.

Supplementary table 7

Intra-individual stability of eGFR decline ≥5 ml/min/1.73m^2/year for different eGFR-cohorts in the PROVALID study over time

	eGFR decline ≥5ml/min/1.73m^2/year			
	2nd year of FU	3rd year of FU	4th year of FU	5th year of FU
eGFR < 60 ml/min (n=149)				
FU2 (n/%)	18 (100)	**9 (60.0)**	**3 (16.7)**	**1 (5.6)**
FU3 (n/%)	9 (69.2)	13 (100)	**5 (38.5)**	**2 (15.4)**
FU4 (n/%)	3 (60.0)	5 (100)	5 (100)	**2 (40.0)**
FU5 (n/%)	1 (50.0)	2 (100)	2 (100)	2 (100)
eGFR ≥ 60 ml/min (n=711)				
FU2 (n/%)	270 (100)	**160 (59.3)**	**109 (40.4)**	**78 (28.9)**
FU3 (n/%)	160 (82.9)	193 (100)	**114 (59.1)**	**78 (40.4)**
FU4 (n/%)	109 (76.8)	114 (80.3)	142 (100)	**85 (59.9)**
FU5 (n/%)	78 (75.0)	78 (75.0)	85 (81.7)	105 (100)

FU: follow up; bold numbers: Number of individuals (%) persistently meeting the definition of eGFR decline over time.

Supplementary table 8

Intra-individual stability of an eGFR decline ≥30% for different urine albumin-to-creatinine ratios (UACR) cohorts in the PROVALID study over time

	eGFR decline ≥30% from baseline until				
	1st year of FU	2nd year of FU	3rd year of FU	4th year of FU	5th year of FU
UACR < 30 mg/g (n=691)					
FU1 (n/%)	16 (100)	**4 (25.0)**	**3 (18.8)**	**3 (18.8)**	**3 (18.8)**
FU2 (n/%)	4 (10.8)	37 (100)	**19 (51.4)**	**13 (35.1)**	**10 (27.0)**
FU3 (n/%)	6 (12.2)	19 (38.8)	49 (100)	**30 (61.2)**	**22 (44.9)**
FU4 (n/%)	10 (15.4)	17 (26.2)	30 (46.2)	65 (100)	**33 (50.8)**
FU5 (n/%)	9 (10.6)	19 (22.4)	31 (36.5)	33 (38.8)	85 (100)
UACR ≥ 30 mg/g (n=169)					
FU1 (n/%)	9 (100)	**2 (22.2)**	**1 (11.1)**	**1 (11.1)**	**1 (11.1)**
FU2 (n/%)	2 (11.1)	18 (100)	**10 (55.6)**	**7 (38.9)**	**6 (33.3)**
FU3 (n/%)	2 (8.3)	10 (41.7)	24 (100)	**14 (58.3)**	**11 (45.8)**
FU4 (n/%)	5 (17.2)	9 (31.0)	14 (48.3)	29 (100)	**20 (69.0)**
FU5 (n/%)	5 (15.6)	13 (40.6)	13 (40.6)	20 (62.5)	32 (100)

FU: follow up; bold numbers: Number of individuals (%) persistently meeting the definition of eGFR decline over time.

Supplementary table 9

Intra-individual stability of an eGFR decline ≥5 ml/min/1.73m^2/year for different urine albumin-to-creatinine ratios (UACR) subgroups in the PROVALID study over time

	eGFR decline ≥5ml/min/1.73m^2/year			
	2nd year of FU	3rd year of FU	4th year of FU	5th year of FU
UACR < 30 mg/g (n=691)				
FU2 (n/%)	227 (100)	**128 (56.4)**	**83 (36.6)**	**57 (25.1)**
FU3 (n/%)	128 (81.1)	158 (100)	**90 (57.0)**	**57 (36.1)**
FU4 (n/%)	83 (74.8)	90 (81.1)	111 (100)	**64 (57.7)**
FU5 (n/%)	57 (71.3)	57 (71.3)	64 (80.0)	80 (100)
UACR ≥ 30 mg/g (n=169)				
FU2 (n/%)	61 (100)	**41 (67.2)**	**29 (47.5)**	**22 (36.1)**
FU3 (n/%)	41 (85.4)	48 (100)	**29 (60.4)**	**23 (47.9)**
FU4 (n/%)	29 (80.6)	29 (80.6)	36 (100)	**23 (63.9)**
FU5 (n/%)	22 (84.6)	23 (88.5)	23 (88.5)	26 (100)

FU: follow up; bold numbers: Number of individuals (%) persistently meeting the definition of eGFR decline over time.

Supplementary table 10a:

Characteristics of prognostic biomarker studies using a two point-method to describe eGFR decline

**Reference**	**N**	**Follow-up (months)**	**Baseline eGFR (ml/min /1.73m²)**	**Definition of kidney function decline**
Chung et al [1]	676	48	83	eGFR decline ≥ 25 %
Peters et al [2]	345	48	80.6	- ≥30 % eGFR decline<br>- incident eGFR < 60<br>- GFR-decline ≥5ml/min/ 1,73m²/year<br>- “rapid decline”: - 4 ml/min/1,73m²/year
Mise et al [3]	675	48	71.4	eGFR decline ≥ 30 % or incident dialysis
Lin et al [4]	516	112	84.3	eGFR decline ≥ 25 %
Velho et al [5]	3101	72	79.2	Doubling of sCr or ESRD
Fernandez-Juarez et al [6]	103	32	47	50% increase in sCr (increase had to be confirmed after 1 month) or incident ESRD
Chang et al [7]	2367	55.2	67.3	Stable: CKD stage stable<br>Regression: change of CKD stage downwards<br>Progression: change of CKD stage upwards
Von Scholten et al [8]	177	58.8	89.8	eGFR decline > 30 %
Nadkarni et al [9]	380	60	88.6	≥ 40 % vs. ≤ 10 % eGFR decline (had to be confirmed in 2 or more visits, at least 3 months apart)
Satirapoj et al [10]	303	12.3	50	≥ 25 % eGFR decline per year
Saulnier et al [11]	1135	52.8	76	≥ 40 % eGFR decline from baseline<br>Rapid: ≥5 ml/min/1,73m²/year (absolute GFR slopes determined by linear regression

				(at least 3 measures, at least 6 months between first and last)<br>Annual GFR trajectory<br>Global pattern of absolute annual eGFR decline
Jenks et al [12]	701	80.4	86	Two eGFR values < 60 ml/min at least 3 months apart with a > 25 % decline
Tan et al [13]	1320	108	84.5	Doubling of sCR or incident ESRD
Fountoulakis et al [14]	101	108	90.7	> 50 % decline in GFR (linear regression model of time on GFR was created and the slope of the regression line was used to estimate the patient's changes of eGFR over time)
Fan et al [15]	80	24	115	Doubling of sCr or ESRD or renal death
Hanai et al [16]	7033	66	69.9	≥ 30 % decrease in eGFR (at least for 3 months) or ESRD<br>≥ 50 % decrease in eGFR or ESRD
Rotbain Curovic et al [17]	192	73.2	89	> 30 % decline in eGFR
Bhensdadia et al [18]	204	72	85	≥ 50 % increase in sCR
Chauhan et al [19]	1245	55.2	74.3	> 40 % decline in eGFR or ESRD
Colombo et al [20]	840	80.2	55.1	> 20 % decline in eGFR

Supplementary table 10b:

Characteristics of prognostic biomarker studies using a slope calculation to describe eGFR decline

Reference	N	Follow-up (months)	Baseline eGFR (ml/min /1.73m^2)	Definition of kidney function decline
Peters et al [2]	345	48	80.6	- GFR-decline ≥5ml/min/ 1,73m^2/year<br>- "rapid decline": - 4 ml/min/1,73m^2/year
Pena et al [21]	82	48	77.9	Accelerated decline: ≥3ml/min/1,73m^2/year
Solini et al [22]	286	36	85	eGFR decline > 10 ml/min/1,73m^2
Liu et al [23]	422	48	80.7	Progression: ≥ 3ml/min/1,73m^2/year decline (at least 3 measures, at least 1 year follow-up)<br>Stable: ± 2 ml/min/1,73m^2/year (at least 5 years of follow-up)
Saulnier et al [11]	1135	52.8	76	Rapid: ≥5 ml/min/1,73m^2/year (absolute GFR slopes determined by linear regression (at least 3 measures, at least 6 months between first and last)<br>Annual GFR trajectory<br>Global pattern of absolute annual eGFR decline
Bhensdadia et al [18]	204	72	85	minus 3,3 % eGFR per year
Nielsen et al [24]	177	42	90	Rate of decline ml/min/1,73m^2/year
Yamamoto et al [25]	161	24	89.6	≥3ml/min/year averaged over 2 years
Chou et al [26]	140	20.4	86.4	eGFR decline rate = $\frac{eGFR_{last}-eGFR_{baseline}}{eGFR_{baseline} \times \text{follow-up}}$
Boertien et al [27]	756	78	65	Annual change in log transformed eGFR were calculated from the slope of the regression line

				through all available eGFR values (at least 3 values, median 6 values)
Narayanan et al [28]	436	96	72	Longitudinal change in eGFR
Agarwal et al [29]	67	22	43	- Slope progression based on inspection<br>- ESRD
Heinzel et al [30]	481	24	84	Progression: 4-5th quintile of eGFR slope
Mayer et al [31]	1765	48	49.2	Annual eGFR loss

References:

1 Chung, H. F. *et al.* Association of n-3 polyunsaturated fatty acids and inflammatory indicators with renal function decline in type 2 diabetes. *Clinical nutrition* **34**, 229-234, doi:10.1016/j.clnu.2014.02.009 (2015).

2 Peters, K. E. *et al.* Identification of Novel Circulating Biomarkers Predicting Rapid Decline in Renal Function in Type 2 Diabetes: The Fremantle Diabetes Study Phase II. *Diabetes care* **40**, 1548-1555, doi:10.2337/dc17-0911 (2017).

3 Mise, K. *et al.* Identification of Novel Urinary Biomarkers for Predicting Renal Prognosis in Patients With Type 2 Diabetes by Glycan Profiling in a Multicenter Prospective Cohort Study: U-CARE Study 1. *Diabetes care* **41**, 1765-1775, doi:10.2337/dc18-0030 (2018).

4 Lin, J., Hu, F. B., Mantzoros, C. & Curhan, G. C. Lipid and inflammatory biomarkers and kidney function decline in type 2 diabetes. *Diabetologia* **53**, 263-267, doi:10.1007/s00125-009-1597-z (2010).

5 Velho, G. *et al.* Plasma copeptin and renal outcomes in patients with type 2 diabetes and albuminuria. *Diabetes care* **36**, 3639-3645, doi:10.2337/dc13-0683 (2013).

6 Fernandez-Juarez, G. *et al.* 25 (OH) vitamin D levels and renal disease progression in patients with type 2 diabetic nephropathy and blockade of the renin-angiotensin system. *Clinical journal of the American Society of Nephrology : CJASN* **8**, 1870-1876, doi:10.2215/CJN.00910113 (2013).

7 Chang, Y. H. *et al.* Serum uric acid level as an indicator for CKD regression and progression in patients with type 2 diabetes mellitus-a 4.6-year cohort study. *Diabetes/metabolism research and reviews* **32**, 557-564, doi:10.1002/dmrr.2768 (2016).

8 von Scholten, B. J. *et al.* Urinary biomarkers are associated with incident cardiovascular disease, all-cause mortality and deterioration of kidney function in type 2 diabetic patients with microalbuminuria. *Diabetologia* **59**, 1549-1557, doi:10.1007/s00125-016-3937-0 (2016).

9 Nadkarni, G. N. *et al.* Association of Urinary Biomarkers of Inflammation, Injury, and Fibrosis with Renal Function Decline: The ACCORD Trial. *Clinical journal of the*

American Society of Nephrology : CJASN **11**, 1343-1352, doi:10.2215/CJN.12051115 (2016).

10 Satirapoj, B., Aramsaowapak, K., Tangwonglert, T. & Supasyndh, O. Novel Tubular Biomarkers Predict Renal Progression in Type 2 Diabetes Mellitus: A Prospective Cohort Study. *Journal of diabetes research* **2016**, 3102962, doi:10.1155/2016/3102962 (2016).

11 Saulnier, P. J. *et al.* Association of Circulating Biomarkers (Adrenomedullin, TNFR1, and NT-proBNP) With Renal Function Decline in Patients With Type 2 Diabetes: A French Prospective Cohort. *Diabetes care* **40**, 367-374, doi:10.2337/dc16-1571 (2017).

12 Jenks, S. J. *et al.* Cardiovascular disease biomarkers are associated with declining renal function in type 2 diabetes. *Diabetologia* **60**, 1400-1408, doi:10.1007/s00125-017-4297-0 (2017).

13 Tan, K. C. B. *et al.* Galectin-3 is independently associated with progression of nephropathy in type 2 diabetes mellitus. *Diabetologia* **61**, 1212-1219, doi:10.1007/s00125-018-4552-z (2018).

14 Fountoulakis, N., Maltese, G., Gnudi, L. & Karalliedde, J. Reduced Levels of Anti-Ageing Hormone Klotho Predict Renal Function Decline in Type 2 Diabetes. *The Journal of clinical endocrinology and metabolism* **103**, 2026-2032, doi:10.1210/jc.2018-00004 (2018).

15 Fan, Y. *et al.* Expression of Endothelial Cell Injury Marker Cd146 Correlates with Disease Severity and Predicts the Renal Outcomes in Patients with Diabetic Nephropathy. *Cellular physiology and biochemistry : international journal of experimental cellular physiology, biochemistry, and pharmacology* **48**, 63-74, doi:10.1159/000491663 (2018).

16 Hanai, K. *et al.* Effects of uric acid on kidney function decline differ depending on baseline kidney function in type 2 diabetic patients. *Nephrology, dialysis, transplantation : official publication of the European Dialysis and Transplant Association - European Renal Association*, doi:10.1093/ndt/gfy138 (2018).

17 Rotbain Curovic, V. *et al.* Urinary tubular biomarkers as predictors of kidney function decline, cardiovascular events and mortality in microalbuminuric type 2 diabetic patients. *Acta diabetologica*, doi:10.1007/s00592-018-1205-0 (2018).

18 Bhensdadia, N. M. *et al.* Urine haptoglobin levels predict early renal functional decline in patients with type 2 diabetes. *Kidney international* **83**, 1136-1143, doi:10.1038/ki.2013.57 (2013).

19 Chauhan, K. *et al.* Plasma endostatin predicts kidney outcomes in patients with type 2 diabetes. *Kidney Int* **95**, 439-446, doi:10.1016/j.kint.2018.09.019 (2019).

20 Colombo, M. *et al.* Serum kidney injury molecule 1 and beta2-microglobulin perform as well as larger biomarker panels for prediction of rapid decline in renal function in type 2 diabetes. *Diabetologia* **62**, 156-168, doi:10.1007/s00125-018-4741-9 (2019).

21 Pena, M. J. *et al.* A panel of novel biomarkers representing different disease pathways improves prediction of renal function decline in type 2 diabetes. *PloS one* **10**, e0120995, doi:10.1371/journal.pone.0120995 (2015).

22 Solini, A. *et al.* Prediction of Declining Renal Function and Albuminuria in Patients With Type 2 Diabetes by Metabolomics. *The Journal of clinical endocrinology and metabolism* **101**, 696-704, doi:10.1210/jc.2015-3345 (2016).

23 Liu, J. J., Liu, S., Wong, M. D., Gurung, R. L. & Lim, S. C. Urinary Haptoglobin Predicts Rapid Renal Function Decline in Asians With Type 2 Diabetes and Early Kidney Disease.

The Journal of clinical endocrinology and metabolism **101**, 3794-3802, doi:10.1210/jc.2016-2094 (2016).
24 Nielsen, S. E. *et al.* Tubular markers are associated with decline in kidney function in proteinuric type 2 diabetic patients. *Diabetes research and clinical practice* **97**, 71-76, doi:10.1016/j.diabres.2012.02.007 (2012).
25 Yamamoto, C. M. *et al.* Uromodulin mRNA from Urinary Extracellular Vesicles Correlate to Kidney Function Decline in Type 2 Diabetes Mellitus. *American journal of nephrology* **47**, 283-291, doi:10.1159/000489129 (2018).
26 Chou, K. M., Lee, C. C., Chen, C. H. & Sun, C. Y. Clinical value of NGAL, L-FABP and albuminuria in predicting GFR decline in type 2 diabetes mellitus patients. *PloS one* **8**, e54863, doi:10.1371/journal.pone.0054863 (2013).
27 Boertien, W. E. *et al.* Copeptin, a surrogate marker for arginine vasopressin, is associated with declining glomerular filtration in patients with diabetes mellitus (ZODIAC-33). *Diabetologia* **56**, 1680-1688, doi:10.1007/s00125-013-2922-0 (2013).
28 Narayanan, R. P. *et al.* IGFBP2 is a biomarker for predicting longitudinal deterioration in renal function in type 2 diabetes. *Endocrine connections* **1**, 95-102, doi:10.1530/EC-12-0053 (2012).
29 Agarwal, R. *et al.* A prospective study of multiple protein biomarkers to predict progression in diabetic chronic kidney disease. *Nephrology, dialysis, transplantation : official publication of the European Dialysis and Transplant Association - European Renal Association* **29**, 2293-2302, doi:10.1093/ndt/gfu255 (2014).
30 Heinzel, A. *et al.* Validation of Plasma Biomarker Candidates for the Prediction of eGFR Decline in Patients With Type 2 Diabetes. *Diabetes Care* **41**, 1947-1954, doi:10.2337/dc18-0532 (2018).
31 Mayer, G. *et al.* Systems Biology-Derived Biomarkers to Predict Progression of Renal Function Decline in Type 2 Diabetes. *Diabetes care* **40**, 391-397, doi:10.2337/dc16-2202 (2017).
